# Decoding protein signaling networks in microvascular angina: the multi-target biochemical actions of traditional Chinese medicine

**DOI:** 10.3389/fmolb.2026.1908353

**Published:** 2026-10-13

**Authors:** Yingkai Duan, Kaifeng Yu, Jing Li, Yuhan Ao, Han Li, Ning Liu, Mingxue Zhang

**Affiliations:** 1 First Clinical College, Liaoning University of Traditional Chinese Medicine, Shenyang, Liaoning, China; 2 The Affiliated Hospital of Liaoning University of Traditional Chinese Medicine, Shenyang, Liaoning, China; 3 Liaoning University of Traditional Chinese Medicine, Shenyang, Liaoning, China; 4 Shenyang Health and Wellness Service Center, Shenyang, Liaoning, China

**Keywords:** ANOCA, coronary microcirculation disorders, endothelial dysfunction, microvascular remodeling, molecular mechanisms, oxidative stress, signaling networks, traditional Chinese medicine

## Abstract

Although angina pectoris is among the most common conditions in cardiology, a substantial proportion of symptomatic patients exhibit persistent ischemia without flow-limiting epicardial coronary artery disease—termed ANOCA (angina with nonobstructive coronary arteries). Accumulating evidence underscores coronary microvascular dysfunction as central to ANOCA’s pathophysiology, involving molecular mechanisms such as endothelial dysfunction, oxidative stress, vascular inflammation, impaired vasodilation, microvascular remodeling, metabolic disturbance, and reduced coronary flow reserve. These interconnected pathways challenge the efficacy of single-target therapies. Traditional Chinese medicine (TCM), with its multi-target systems-based approach, offers a promising therapeutic model. Preclinical and clinical studies reveal that representative TCM formulas and bioactive compounds preserve endothelial integrity, suppress pro-inflammatory cytokines, attenuate oxidative injury, restore mitochondrial and metabolic homeostasis, stimulate angiogenesis, and improve myocardial perfusion. Such effects align with the molecular complexity of ANOCA, suggesting TCM could complement conventional cardiovascular treatments. This review synthesizes current understanding of coronary microvascular pathobiology, traces the evolution of microvascular-protective therapeutic concepts, and evaluates TCM’s mechanistic and clinical evidence base. While findings highlight TCM’s therapeutic potential, translational limitations persist—including heterogeneity in diagnostic criteria, variability in herbal formulations, methodological inconsistencies, and gaps in robust evidence. Integrating mechanistic, clinical, and translational data, this work delineates the scientific foundation, therapeutic promise, and future research directions for TCM in ANOCA-related coronary microcirculation disorders, advocating for standardized, mechanism-driven investigations to enable evidence-based integration into cardiovascular care.

## 1 Introduction

Angina with non-obstructive coronary arteries (ANOCA) is one of the most consequential reframings in contemporary cardiology. Between one-third and one-half of patients with recurrent angina, exertional chest tightness, or objective evidence of ischemia have no obstructive epicardial disease at coronary angiography (Ahmad et al., 2023; Boerhout et al., 2023; Feldman et al., 2022; Gatti et al., 2022; Hwang et al., 2023; Maayah et al., 2024). These patients were once labeled as having “atypical” or “low-risk” chest pain, but it is now established that functional and structural abnormalities of the coronary microvasculature—impaired endothelium-dependent and -independent vasodilation, microvascular spasm, capillary rarefaction, inflammation, and oxidative injury—can produce ischemia in the absence of obstructive disease (Crea et al., 2022; Pepine, 2023; Sucato et al., 2024; Tudurachi et al., 2024). ANOCA is associated with persistent symptoms, reduced functional capacity, repeated healthcare utilization, and, in defined subgroups, increased long-term cardiovascular risk (de Souza et al., 2022; Samuels et al., 2023).

The pathobiology that underlies ANOCA is not a single lesion but a set of interacting microvascular processes. Endothelial nitric oxide synthase (eNOS) becomes dysfunctional or uncoupled under oxidative stress, producing reactive oxygen species rather than nitric oxide; NADPH oxidase activity rises and feeds a redox-inflammatory loop centered on NF-κB signaling; mitochondrial bioenergetic injury reduces endothelial repair capacity; and chronic remodeling produces capillary rarefaction and impaired angiogenic response (Crea et al., 2022; Del Buono et al., 2021; Galante et al., 2025; Kei et al., 2023; Kunadian et al., 2020; Parlati et al., 2025; Scarica et al., 2025; Figure 1). These processes are not independent. Endothelial dysfunction amplifies oxidative stress, which sustains inflammation, which in turn drives further endothelial injury and remodeling. Pharmacological interruption at one node of this network is, in principle, insufficient when other nodes remain active.

**FIGURE 1:**
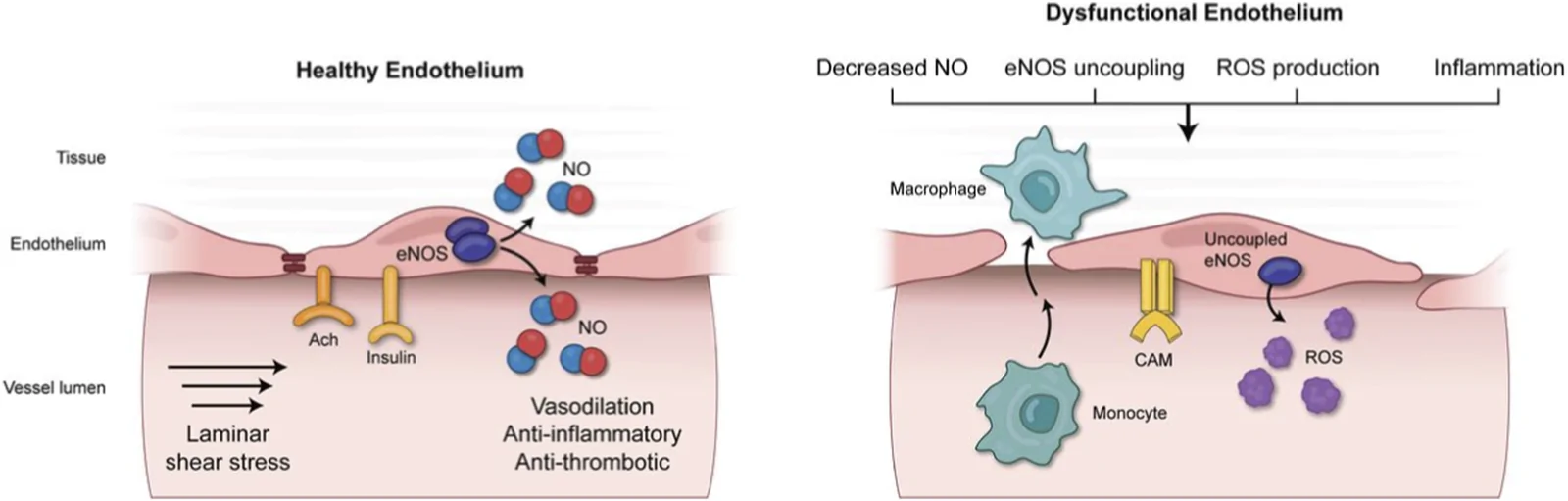
Nitric oxide (NO) signalling in healthy and dysfunctional endothelium. The healthy endothelium (left) maintains normal vascular function: tight junctions are present between endothelial cells; the cells exhibit an anti-inflammatory and antithrombotic phenotype; and laminar shear stress, insulin, and acetylcholine stimulate endothelial nitric oxide synthase (eNOS) to produce NO, which mediates vasodilation. The dysfunctional endothelium (right) shows reduced NO production, eNOS uncoupling with increased reactive oxygen species (ROS) generation, increased expression of cell adhesion molecules (CAMs), and enhanced monocyte and macrophage infiltration into the vessel wall. The net result is a reduction in NO and an increase in ROS that promotes the development and progression of microvascular dysfunction.

This network architecture has direct therapeutic implications. The PI3K/Akt-eNOS axis maintains endothelial homeostasis; the AMPK and SIRT1 pathways link metabolic stress to redox balance; the HIF-1α–VEGF axis governs adaptive angiogenesis; NF-κB integrates inflammatory input from cytokines, oxidized lipids, and endothelial activation; and the Nrf2/HO-1 axis coordinates antioxidant defense. These pathways operate in parallel and exhibit cross-talk: metabolic stress activates AMPK, AMPK regulates eNOS phosphorylation, eNOS uncoupling generates ROS, ROS activate NF-κB, and NF-κB amplifies endothelial inflammation (Figure 2). A single-target intervention—for example, an eNOS recoupler or an NF-κB inhibitor—addresses one node but does not block the feedback that sustains the system. Combination pharmacotherapy aimed at multiple network nodes is a reasonable theoretical response. Traditional Chinese medicine (TCM) is one historically established system of multi-component therapy in which multiple bioactive constituents act on multiple network nodes simultaneously, and is therefore worth examining as a candidate model for this disease class.

**FIGURE 2:**
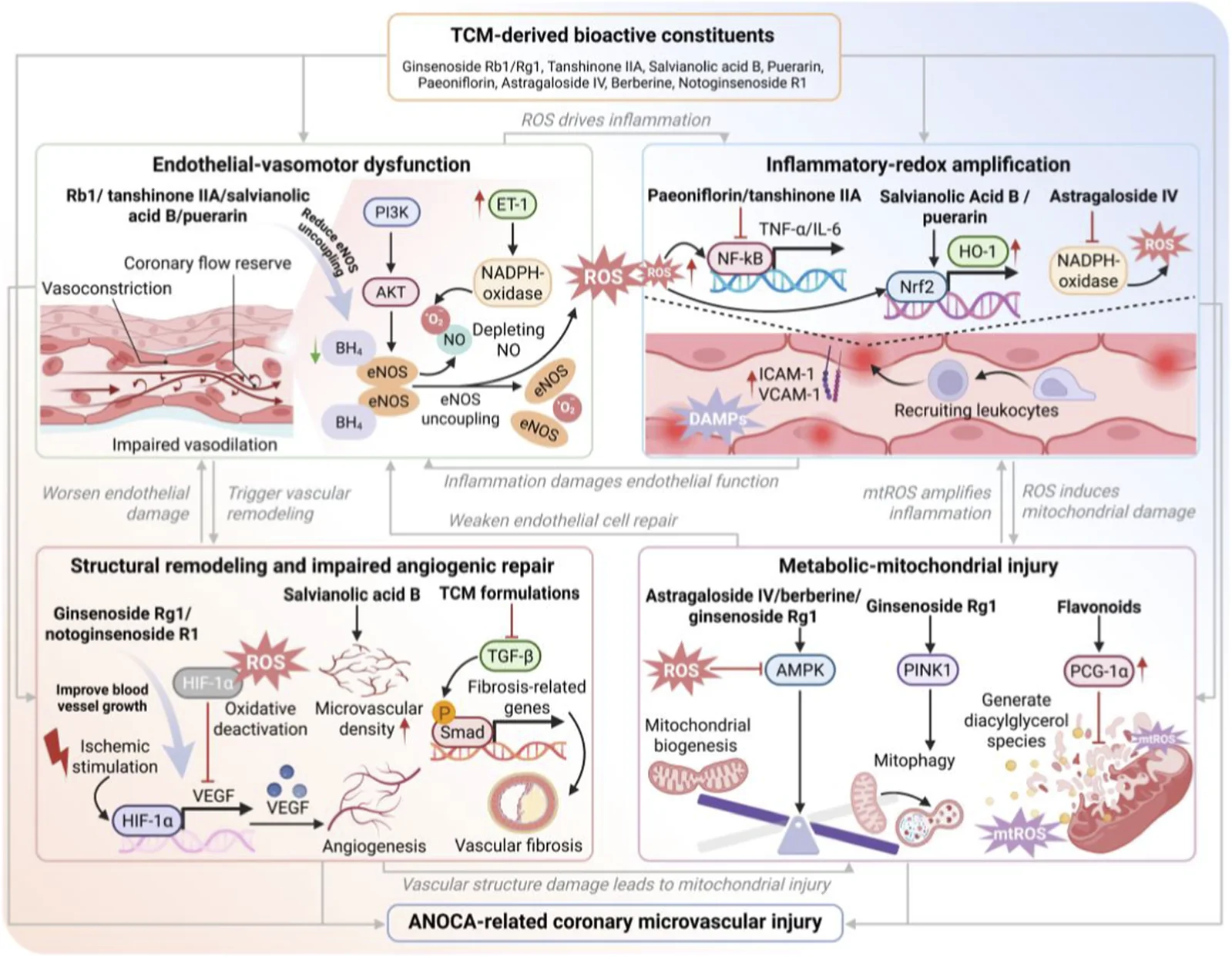
Schematic of four-module crosstalk in ANOCA and multi-target TCM actions.

Conventional cardiovascular pharmacotherapy was developed primarily against obstructive disease—slowing heart rate, dilating epicardial conduit arteries, reducing afterload, and preventing thrombosis (Barrios and Escobar, 2021; Knuuti et al., 2020). These therapies remain useful, but they were not designed for the integrated endothelial, inflammatory, metabolic, and microcirculatory abnormalities that characterize ANOCA. The therapeutic gap is now widely acknowledged in cardiovascular medicine (Crea et al., 2022; Del Buono et al., 2021; Kei et al., 2023; Scarica et al., 2025).

A typical TCM formulation contains multiple plant-derived, mineral, or animal-derived components whose constituents act on a wide range of molecular targets. Modern systems pharmacology has begun to make this multi-target structure tractable: network pharmacology methods can map specific compounds to specific targets, and protein-protein interaction networks can identify the disease modules these compounds reach (Hua et al., 2025; McChord et al., 2023; Yang et al., 2022). Experimental and clinical studies have reported that representative TCM formulations—including Shexiang Baoxin Pill, Tongxinluo Capsule, Tongmai Jiangtang Capsule, Shexiang Tongxin Dropping Pill, and Compound Danshen Dropping Pill—affect coronary flow reserve, endothelial function, inflammatory and oxidative biomarkers, myocardial perfusion, and symptom burden (Zhou et al., 2024; Zuo et al., 2021). Reported mechanisms include increased NO bioavailability, reduced endothelin-mediated vasoconstriction, suppression of NF-κB and downstream inflammatory mediators, attenuation of NADPH oxidase activity, modulation of PI3K/Akt, AMPK, and HIF-1α/VEGF signaling, preservation of mitochondrial integrity, and promotion of adaptive angiogenesis (Ge et al., 2025; Liu et al., 2025; Yu et al., 2022; Figure 3).

**FIGURE 3:**
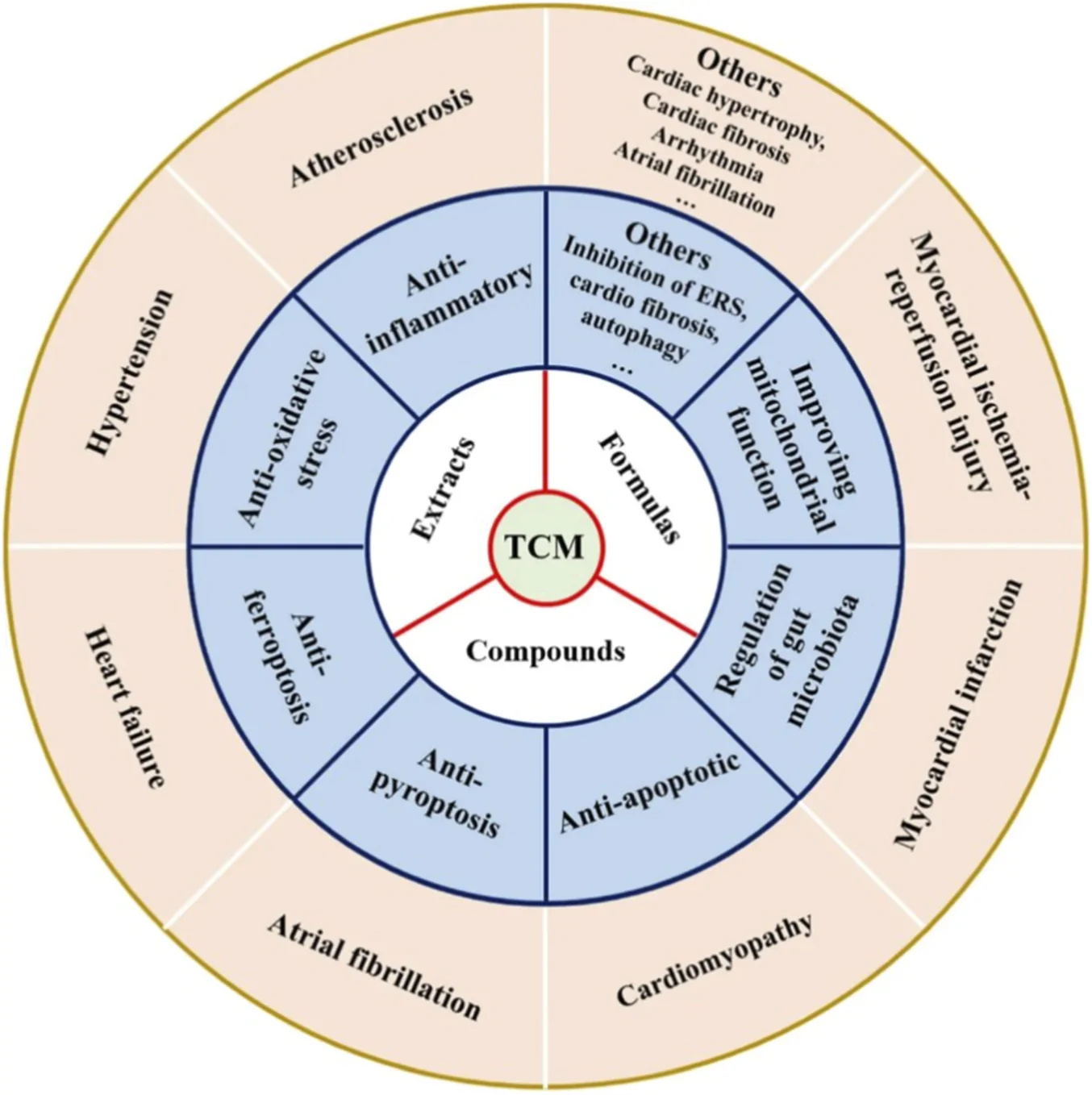
Conceptual overview of the multi-target actions of traditional Chinese medicine (TCM) on cardiovascular disease, integrating extracts, formulas, and isolated bioactive compounds. The inner ring summarizes principal mechanistic actions (anti-oxidative stress, anti-inflammatory, anti-ferroptosis, anti-pyroptosis, anti-apoptotic, regulation of gut microbiota, improvement of mitochondrial function, and other actions including inhibition of endoplasmic reticulum stress and modulation of cardio-fibrosis and autophagy). The outer ring summarizes the principal cardiovascular disease categories addressed (atherosclerosis, hypertension, heart failure, atrial fibrillation, cardiomyopathy, myocardial infarction, myocardial ischemia-reperfusion injury, and others such as cardiac hypertrophy, fibrosis, and arrhythmia). This figure is intended as a mechanism-map for hypothesis generation and does not imply ANOCA-specific clinical efficacy. Abbreviations: ANOCA, angina with non-obstructive coronary arteries; TCM, traditional Chinese medicine. Adapted with reference to Dai et al. (2024).

These reports require evidence-calibrated interpretation. The current evidence base is uneven. A single phase IV randomized controlled trial—the phase IV ANOCA trial of Shexiang Baoxin Pill (n = 239) — provides directc phenotype-defined clinical evidence (He et al., 2025). Other supportive evidence is drawn from clinical trials in stable coronary artery disease, microvascular angina, or diabetic vascular complications, from animal models of myocardial ischemia–reperfusion or microvascular injury, or from *in vitro* and network-pharmacology studies. Conflating these tiers—speaking of “demonstrated efficacy” when the actual evidence is preclinical, or claiming microvascular protection from cardiomyocyte data—has been a recurrent problem in the TCM literature. The present review therefore explicitly stratifies the evidence by tier and calibrates the strength of every claim accordingly.

Several recent reviews have addressed TCM for cardiovascular disease at a general level (Hao et al., 2015; Layne and Ferro, 2017), or have summarized individual formulations such as Shexiang Baoxin Pill (Lu et al., 2018), Tongxinluo (Xin et al., 2024), or Compound Danshen Dropping Pill. The present review differs in three respects. First, ANOCA—rather than coronary microvascular dysfunction in general or chronic coronary disease as a whole—is taken as the explicit clinical anchor, and the boundary between ANOCA-specific and microvascular-adjacent evidence is maintained throughout. Second, mechanism mapping is performed at the bioactive-compound level wherever possible, with each constituent traced to a defined molecular target rather than to a formula-level effect summary. Third, an explicit five-tier evidence hierarchy (Section 2.2) is applied throughout, so that biologically plausible findings from network pharmacology, cell models, and animal studies are not equated with phenotype-defined clinical efficacy. The intended contribution is an evidence-calibrated, mechanism-anchored synthesis rather than a broad descriptive overview.

The aims of this review are to (i) define the molecular and cellular substrate of coronary microvascular dysfunction underlying ANOCA, with emphasis on the mechanisms most relevant to pharmacological intervention; (ii) examine the extent to which the multi-target pharmacology of representative TCM formulations is mechanistically congruent with that substrate; (iii) grade the evidence supporting individual formulations against an explicit hierarchy; and (iv) identify the translational steps required before any of these formulations can be incorporated into evidence-based ANOCA management.

## 2 Materials and methods

### 2.1 Study design and reporting framework

This work is a structured narrative review, not a systematic review. This review does not include a formal risk-of-bias assessment, a meta-analytic summary, or a quantitative synthesis, and does not aspire to be exhaustive of every TCM-related publication. The aim is to integrate the molecular substrate of coronary microvascular dysfunction in ANOCA with the mechanistic and clinical evidence available for representative TCM formulations, within an explicit evidence hierarchy. The protocol was not pre-registered, and we do not claim equivalence with a registered systematic review.

We systematically searched PubMed/MEDLINE, Web of Science, Scopus, Embase, and the Cochrane Library from inception to 1 January 2026. Only full-text peer-reviewed publications were eligible; no language restriction was applied at the initial retrieval stage, but final inclusion required English-language full text. To accommodate the heterogeneous terminology in this field, we developed a search strategy structured around three conceptual blocks: (1) disease-related terms (“angina with non-obstructive coronary arteries,” “ANOCA,” “INOCA,” “microvascular angina,” “coronary microvascular dysfunction,” “coronary flow reserve,” “coronary endothelial dysfunction”); (2) intervention-related terms (“traditional Chinese medicine,” “Chinese herbal medicine,” “Chinese patent medicine,” “herbal formula,” “Shexiang Baoxin Pill,” “Tongxinluo,” “Tongmai Jiangtang Capsule,” “Danshen,” “Shexiang Tongxin Dropping Pills”); and (3) mechanism- or outcome-related terms (“endothelial function,” “oxidative stress,” “inflammation,” “angiogenesis,” “microvascular remodeling,” “vasomotor regulation,” “myocardial perfusion,” “vascular protection”). Boolean operators (AND/OR) were used to combine terms, with database-specific syntax adaptations. Reference lists of eligible studies and relevant systematic reviews were hand-searched for additional records. Two independent reviewers performed title/abstract screening and full-text eligibility assessment. Disagreements were resolved by discussion, with arbitration by a senior author when consensus could not be reached. The final included studies were thematically synthesised according to the four microvascular biology modules (endothelial-vasomotor, inflammatory-redox, metabolic-mitochondrial, and structural-remodelling) to maintain conceptual coherence between mechanistic evidence and clinical observations. The specific inclusion and exclusion criteria are shown in Table 1.

**TABLE 1:** Inclusion and exclusion criteria for study selection.

Inclusion criteria	Exclusion criteria
Original peer-reviewed studies evaluating traditional Chinese medicine, Chinese herbal medicine, Chinese patent medicine, or named TCM-based compound interventions	Editorials, commentaries, letters, opinion pieces, conference abstracts without full text, dissertations, and non-peer-reviewed publications
Studies focused on ANOCA, INOCA, coronary microcirculation disorders, coronary microvascular dysfunction, microvascular angina, or non-obstructive ischemic syndromes with clear microvascular relevance	Studies focused only on obstructive epicardial coronary artery disease without microcirculatory or endothelial relevance
Clinical, translational, animal, or *in vitro* studies with outcomes related to endothelial function, inflammation, oxidative stress, angiogenesis, vasomotor regulation, coronary flow reserve, myocardial perfusion, or microvascular remodeling	Studies with no meaningful assessment of coronary microvascular biology, endothelial pathways, perfusion outcomes, or related mechanistic endpoints
Studies reporting mechanistic, physiological, or clinically relevant cardiovascular effects of TCM interventions	Studies in which the intervention was not part of traditional Chinese medicine or could not be reasonably classified within a TCM framework
Full-text articles published in peer-reviewed journals	Duplicate reports, incomplete datasets, or multiple publications from the same study population where a more complete version was available
Articles written in English or otherwise accessible for accurate scientific evaluation by the authors	Articles lacking sufficient methodological detail, outcome clarity, or interpretability for evidence synthesis

### 2.2 Evidence hierarchy

Because the available evidence spans clinical trials, animal studies, and *in vitro* mechanism work, with markedly different epistemic weight, we applied an explicit five-tier hierarchy throughout the review. Therapeutic language is calibrated to each tier, and the hierarchy is enforced in the discussion of every formulation and constituent.

Tier 1 — Direct ANOCA randomized clinical evidence: randomized controlled trials in patients with formally defined ANOCA, with prespecified microvascular or angina-specific endpoints. Statements based on Tier 1 evidence use language such as “demonstrated” or “established.”

Tier 2 — Microvascular angina or INOCA clinical evidence: randomized or prospective trials in patients with microvascular angina, INOCA, or coronary microvascular dysfunction defined by invasive testing (CFR, IMR, acetylcholine), where the population overlaps with but is not identical to ANOCA. Statements use “supported by” or “associated with.”

Tier 3 — General coronary or angina clinical evidence: randomized or prospective trials in stable coronary artery disease, generic angina, or diabetic cardiovascular complications, where microvascular involvement is biologically plausible but not invasively confirmed. Statements use “consistent with” or “may support.”

Tier 4 — Animal evidence: controlled *in vivo* studies in models of myocardial ischemia–reperfusion, hyperglycemic vascular injury, hypertensive microvascular dysfunction, or angiogenic deficit. Statements use “preclinical evidence suggests” or “*in vivo* studies indicate.”

Tier 5 — *In vitro* and computational evidence: cell-culture studies of endothelial, smooth-muscle, or cardiomyocyte function; isolated-vessel preparations; and network-pharmacology or molecular-docking analyses. Statements use “*in vitro* studies indicate” or “is hypothesized.”

Where evidence for a given claim is mixed across tiers, the lowest applicable tier governs the language. Table 2 summarizes the hierarchy. Systematic reviews and meta-analyses were not assigned an independent tier based on their design. Instead, their classification followed the highest phenotyping standard present in their constituent primary studies. A meta-analysis that included ≥1 ANOCA phenotype-defined RCT was classified at Tier 1 or Tier 2 depending on whether the ANOCA-specific effect was separately extractable. A meta-analysis composed entirely of stable CAD, generic angina, or diabetic cohorts—none of which required invasive CFR, IMR, or acetylcholine testing for entry—was assigned to Tier 3. Where constituent studies spanned multiple tiers, the lowest applicable tier governed the classification, and the cohort phenotypes contributing to each meta-analysis are specified in Section 5.

**TABLE 2:** Evidence hierarchy applied throughout the review.

Tier	Evidence type	Description	Therapeutic language used
1	Direct ANOCA RCT	Randomized controlled trial in patients with formally defined ANOCA, with prespecified microvascular or angina-specific endpoints	“Demonstrated”/“established”
2	Microvascular angina/INOCA RCT	Randomized or prospective trial in microvascular angina, INOCA, or invasively confirmed CMD. Population overlaps with but is not identical to ANOCA.	“Supported by”/“associated with”
3	General coronary clinical evidence	Trial in stable CAD, generic angina, or diabetic cardiovascular complications; microvascular involvement plausible but not invasively confirmed	“Consistent with”/“may support”
4	Animal evidence	*In vivo* studies in models of myocardial ischemia–reperfusion, hyperglycemic vascular injury, hypertensive microvascular dysfunction, or angiogenic deficit	“Preclinical evidence suggests”
5	*In vitro*/computational	Cell-culture studies; isolated-vessel preparations; network-pharmacology or molecular-docking analyses	“In vitro studies indicate”/“is hypothesized”

ANOCA is positioned as the broader clinical diagnosis (symptom-based), whereas INOCA represents a subset of ANOCA in whom objective ischemia is demonstrable. Accordingly, trials enrolling formally defined ANOCA populations (Tier 1) provide evidence applicable to the full spectrum of non-obstructive angina, including both INOCA and non-ischemic ANOCA subgroups. To contextualise the diagnostic reliability of the ANOCA trials included in this review, we summarise the diagnostic approaches reported in the source studies. The phase IV SBP trial (He et al., 2025) enrolled patients with ANOCA and used the Seattle Angina Questionnaire as the primary efficacy endpoint; it does not specify whether invasive coronary function testing (CFR, IMR, or acetylcholine provocation) was required for enrollment. The LESS trial (Liang et al., 2024) enrolled INOCA patients and similarly used SAQ as the primary endpoint. By contrast, the meta-analysis of blood-activating drugs for microvascular angina (Zhong et al., 2020) applied diagnostic criteria of angina-like chest pain plus positive exercise treadmill testing (ST-segment depression ≥0.1 mV), without requiring invasive functional confirmation. For TJC (Wan et al., 2025), the enrolled population was T2DM patients with coronary heart disease, and microvascular phenotyping was not reported. These descriptions are limited by what is reported in the published abstracts; full-text review may reveal additional methodological details.

### 2.3 Data extraction and synthesis

Information extracted from each included study comprised: bibliographic identifiers; study design and sample size; population characteristics; intervention chemistry (named formulation, characterized constituents, dose, route, duration); comparator; primary and secondary outcomes; mechanistic readouts; and reported limitations. Findings were synthesized thematically along the four microvascular biology modules described in Section 3 (endothelial-vasomotor, inflammatory-redox, metabolic-mitochondrial, and structural-remodeling). The synthesis is qualitative; no quantitative meta-analysis was performed.

## 3 Targetable microvascular mechanisms in ANOCA

Coronary microvascular dysfunction in ANOCA is best understood not as a list of independent abnormalities but as a connected network of four interacting modules: endothelial-vasomotor dysfunction, inflammatory-redox amplification, metabolic-mitochondrial injury, and structural remodeling with impaired angiogenic repair. Each module is biologically distinct but mechanistically coupled to the others. The therapeutic implication, developed across the rest of this review, is that meaningful intervention will require simultaneous engagement of multiple modules—a problem to which a multi-component pharmacology may be structurally suited. The molecular targets of each module and their corresponding clinical endpoints are summarized in Table 3.

**TABLE 3:** Molecular targets and their corresponding clinical endpoints in ANOCA.

Module	Molecular target	Corresponding clinical index	Measurement method	Validation status in ANOCA
Endothelial-vasomotor	eNOS/NO bioavailability	Coronary flow reserve (CFR); endothelium-dependent vasodilation	Doppler flow wire; echocardiography; acetylcholine/adenosine challenge	Validated (Tier 1–2)
Endothelial-vasomotor	ET-1	Plasma ET-1 levels	ELISA	Speculative (Tier 3–5)
Inflammatory-redox	NF-κB/cytokines	hs-CRP, IL-6, TNF-α	Serum immunoassay	Validated (Tier 2–3)
Inflammatory-redox	NADPH oxidase/ROS	MDA, SOD, 8-OHdG	Serum/plasma assay	Speculative (Tier 4–5)
Metabolic-mitochondrial	AMPK/metabolic regulation	Fasting glucose, HOMA-IR, lipid profile	Standard biochemical assays	Validated (Tier 3; TJC in diabetic cohorts)
Metabolic-mitochondrial	Mitochondrial function	No direct clinical index (assessed via CFR or PET perfusion as proxy)	CFR; PET myocardial perfusion	Speculative (Tier 4–5)
Structural-remodeling	HIF-1α/VEGF	Circulating VEGF; collateral score; myocardial perfusion imaging	Serum VEGF; angiography; SPECT/PET	Speculative (Tier 4–5)
Structural-remodeling	TGF-β/fibrosis	No direct clinical index (IMR or CFR as functional proxy)	Invasive IMR/CFR measurement	Speculative (Tier 4–5)

### 3.1 Endothelial-vasomotor dysfunction

The coronary microvascular endothelium regulates vasomotor tone, barrier integrity, leukocyte trafficking, and angiogenic repair. In health, endothelial NO synthase (eNOS) generates nitric oxide from L-arginine, with tetrahydrobiopterin (BH4) as cofactor; NO diffuses to vascular smooth muscle, activates soluble guanylate cyclase, and produces vasodilation (Abu-Soud et al., 2025; Krüger-Genge et al., 2019). In ANOCA-related coronary microvascular dysfunction, this axis is disrupted at multiple levels. Reduced BH4 availability or NADPH oxidase-derived superoxide leads to eNOS uncoupling: the enzyme produces superoxide rather than NO, simultaneously depleting NO and amplifying oxidative stress (Daiber et al., 2017; Münzel and Daiber, 2023). Concurrent upregulation of endothelin-1 (ET-1) shifts the vasomotor balance toward constriction (Drożdż et al., 2023; Grego et al., 2025). The resulting picture—impaired endothelium-dependent vasodilation, exaggerated vasoconstrictor response, and reduced coronary flow reserve—is the cardinal functional signature of ANOCA.

Endothelial dysfunction is also a structural insult. Loss of endothelial proliferative capacity, increased apoptosis, and impaired migration reduce the vessel’s capacity for repair (Botts et al., 2021; Naderi-Meshkin and Setyaningsih, 2024). These changes connect endothelial-vasomotor pathology to the remodeling module discussed in Section 3.4.

Therapeutically, this module has been a focus of TCM research because several characterized constituents—most notably ginsenoside Rb1, tanshinone IIA, salvianolic acid B, and puerarin—converge on the eNOS-NO axis, either by enhancing eNOS phosphorylation through PI3K/Akt, by reversing eNOS uncoupling, or by improving L-arginine substrate availability through cationic amino acid transporters. These mechanisms are discussed in Section 5.

### 3.2 Inflammatory-redox amplification

Microvascular inflammation in ANOCA arises from endothelial activation, circulating cytokines (TNF-α, IL-6), and danger-associated molecular patterns released by mechanical or metabolic stress (Balta, 2020; Theofilis et al., 2021). Activated endothelium expresses VCAM-1 and ICAM-1, recruiting leukocytes; NF-κB signaling sustains cytokine output; and chronic low-grade inflammation impairs endothelium-dependent vasodilation independent of structural lesions (Ashokprabhu et al., 2023; Feuer et al., 2022).

Inflammation is mechanistically inseparable from oxidative stress. NADPH oxidases (especially NOX2 and NOX4 in vascular tissue) generate superoxide and hydrogen peroxide; mitochondrial dysfunction adds to the ROS burden; and oxidative modification of BH4 perpetuates eNOS uncoupling (Daiber et al., 2017; Münzel and Daiber, 2023). The redox-inflammatory loop—ROS activates NF-κB, NF-κB amplifies cytokine output, cytokines further activate NADPH oxidase—is a self-sustaining feature of microvascular dysfunction (Theofilis et al., 2021; Yan et al., 2023).

Several TCM constituents target this loop at multiple points: paeoniflorin and tanshinone IIA inhibit NF-κB activation; salvianolic acids and puerarin engage Nrf2/HO-1 antioxidant signaling; and astragaloside IV reduces NADPH oxidase activity. Their relevance to ANOCA microvascular biology is discussed in Section 5.

### 3.3 Metabolic and mitochondrial injury

Coronary microvascular endothelium has high metabolic demand and is sensitive to substrate availability and mitochondrial integrity. In hyperglycemic, dyslipidemic, or insulin-resistant states—common in ANOCA cohorts with cardiometabolic risk factors—endothelial mitochondria show reduced membrane potential, fragmented morphology, and impaired oxidative phosphorylation (Horton and Barrett, 2021; Römer et al., 2021). Mitochondrial ROS production rises, AMPK signaling is suppressed, and the balance between mitochondrial biogenesis and mitophagy is disturbed (Marassi and Fadini, 2023).

Fatty acid oxidation–glycolysis imbalance also contributes. The endothelium normally derives most of its ATP from glycolysis, but lipotoxic conditions and hyperglycemia shift substrate utilization, generate diacylglycerol species that activate protein kinase C, and impair endothelial function through both direct and indirect mechanisms (Horton and Barrett, 2021; Rana et al., 2007). These metabolic insults are reversible in principle but require interventions that act simultaneously on metabolism and on the mitochondrial machinery (Zhao et al., 2020).

Astragaloside IV, berberine, and ginsenoside Rg1 act on AMPK; ginsenoside Rg1 additionally engages PINK1-mediated mitophagy; and several TCM-derived flavonoids influence mitochondrial biogenesis through PGC-1α. Mechanistic detail is presented in Section 5.

### 3.4 Structural remodeling and impaired angiogenic repair

Chronic microvascular dysfunction produces structural change. Capillary rarefaction reduces tissue perfusion capacity; vessel wall thickening from smooth muscle hypertrophy and fibroblast activation increases resistance; and perivascular fibrosis—driven by TGF-β and downstream collagen deposition—further compromises microcirculatory function (Brandt et al., 2021; Jiang et al., 2022). These changes are evident on histology in ANOCA models and on advanced imaging in patients (Collet et al., 2024).

The angiogenic counterweight is impaired. The HIF-1α–VEGF axis, which normally drives compensatory capillary growth in response to ischemic stimuli, is blunted in chronic microvascular disease, in part because of NO deficiency, in part because of oxidative inactivation of HIF-1α stabilizers, and in part because endothelial proliferative capacity itself is reduced (Kloka et al., 2023; Miao et al., 2022).

Ginsenoside Rg1 and notoginsenoside R1 have been reported to enhance HIF-1α-VEGF signaling and to promote endothelial sprouting in preclinical models; salvianolic acid B improves microvascular density in ischemic myocardium; and several TCM formulations reduce perivascular fibrosis through TGF-β-related pathways. These findings are summarized at the formulation level in Section 5.

The four modules above are interlocked. Endothelial dysfunction generates ROS; ROS activate inflammation; inflammation accelerates mitochondrial injury; mitochondrial injury reduces endothelial repair; and structural remodeling reduces the substrate on which functional recovery would otherwise depend. A pharmacological strategy that engages only one module is, on this view, structurally incomplete (Crooijmans and Damman, 2024). The argument for examining TCM as a candidate intervention class is that the chemistry of representative formulations distributes activity across multiple modules in parallel—a property that is examined at the constituent level in the following sections.

## 4 Traditional Chinese medicine as a structured multi-target pharmacology

### 4.1 From multi-target to structured polypharmacology

The phrase “multi-target therapy” is sometimes applied loosely to any intervention that affects more than one biological pathway. In the case of TCM, the multi-target property is structural rather than incidental. A single formulation contains multiple plant- or animal-derived components, each with its own characterized constituents and known molecular targets; classical pairing principles select these components for complementary, rather than redundant, actions. Modern systems pharmacology, applied retrospectively, has confirmed that representative formulations distribute activity across non-overlapping disease modules to a degree that would be difficult to achieve by simple co-administration of single-target drugs (Hua et al., 2025; Li et al., 2025; McChord et al., 2023); emerging machine-learning approaches are extending this multi-target paradigm to drug discovery (Bi et al., 2025).

This distinction is methodologically important. A polypharmacology that emerges by chance from a complex extract is difficult to standardize and to test. A polypharmacology that is structurally defined—with specified bioactive constituents, defined target distribution, and reproducible chemical fingerprinting—is, in principle, testable in the same way as conventional pharmacology. Network pharmacology and systems pharmacology methods make this testability operational by mapping characterized constituents to known targets, identifying the disease modules they reach, and predicting which combinations are biologically rational (Hua et al., 2025; McChord et al., 2023; Yang et al., 2022).

The relevant issue is not whether a formulation touches many targets, but whether its constituents engage functionally coupled nodes within the same pathogenic circuit. For example, a constituent that restores eNOS-dependent vasodilation may be mechanistically reinforced by another constituent that suppresses NF-κB-driven endothelial inflammation, because inflammation and oxidative stress perpetuate eNOS uncoupling and microvascular constriction. In this sense, structured polypharmacology refers to coordinated modulation of interdependent disease modules rather than the mere accumulation of unrelated pharmacological effects; at present, such synergy should be regarded as mechanistically plausible and hypothesis-generating unless supported by formal dose–response or component-combination experiments.

However, this approach should not be interpreted as providing clinical-grade evidence of efficacy. Network pharmacology generates hypotheses about mechanism. Validation requires confirmation in animal studies and subsequently in clinical studies of defined phenotypes. Maintaining this evidence hierarchy is essential to prevent mechanistic plausibility from being interpreted as clinical efficacy; the following sections apply this framework to each formulation.

### 4.2 Syndrome-phenotype correspondence: an exploratory framework

In Chinese medicine, recurrent exertional chest pain is described under the patterns of qi-deficiency with blood stasis, phlegm-turbidity obstructing the chest, and yang-deficiency with cold congealing, among others (Marshall, 2020; Su, 2015). In contemporary TCM cardiology these syndrome patterns are used to select among formulations. Whether they correspond to mechanistically distinct subtypes of ANOCA-related microvascular dysfunction is an open question, and one that this review treats as exploratory rather than established.

Conceptually, blood-stasis patterns approximate impaired perfusion, endothelial dysfunction, and thrombo-inflammatory phenotypes; qi-deficiency patterns approximate diminished cardiovascular reserve and mitochondrial-metabolic dysfunction; phlegm-turbidity approximates the cardiometabolic-inflammatory phenotype seen in obesity, dyslipidemia, and insulin resistance. These mappings are heuristic. No prospective study has formally validated a TCM pattern against invasive measures of coronary microvascular function (CFR, IMR, acetylcholine response). Statements about pattern–phenotype correspondence in this review are therefore labeled hypothesis-generating, and any clinical implication is contingent on prospective validation.

### 4.3 Systems-pharmacology workflow

Where applied rigorously, TCM systems pharmacology (Li et al., 2023) proceeds through a defined workflow: chemical characterization of the formulation by HPLC or LC-MS, identification of bioactive constituents that meet pharmacokinetic filters (oral bioavailability, drug-likeness), prediction of molecular targets via curated databases (DrugBank, TCMSP, BindingDB), construction of a compound–target network, intersection with disease-target sets (DisGeNET, OMIM), and topological analysis of the resulting network to identify hub targets and disease modules (Hua et al., 2025; McChord et al., 2023; Yang et al., 2022; Yuan et al., 2022). The output is a list of testable mechanistic hypotheses, prioritized by network centrality.

When complemented by experimental validation—knockdown or pharmacological inhibition of predicted hub targets, dose–response confirmation of effects on relevant readouts, and animal-model recapitulation—this workflow generates evidence at Tier 4 or Tier 5 of the hierarchy in Section 2.2. It does not, by itself, generate Tier 1 or Tier 2 evidence. Studies that present network-pharmacology output as if it were clinical evidence misuse the method.

## 5 Representative TCM formulations: evidence-weighted synthesis

Five formulations have accumulated the most relevant published evidence for ANOCA-related coronary microvascular biology and form the focus of this section (Xing et al., 2022). Each subsection follows a uniform structure: chemical composition and characterized constituents, mechanistic findings linked to the four modules of Section 3, available clinical evidence with explicit tier, and current limitations. The composition and chemical characterization of each formulation are summarized in Table 4, and compound-level mechanism mapping is summarized in Table 5.

**TABLE 4:** Composition and chemical characterization of representative TCM formulations.

Formulation	Composition	Characterized constituents (relevant to microvascular biology)	Chemical characterization method	Regulatory status
Shexiang Baoxin Pill (SBP)	Moschus; *Panax ginseng* C.A. Mey.; Calculus Bovis Artifactus; *Cinnamomum cassia* Presl; Styrax from *Liquidambar orientalis* Mill.; Bufonis Venenum from *Bufo gargarizans* Cantor; Borneolum Syntheticum	Ginsenoside Rb1, Rg1; muscone; bufalin, cinobufagin; cinnamic acid; borneol; >70 non-volatile + 40 volatile constituents	HPLC, GC-MS	Chinese Pharmacopoeia 2015
Tongxinluo Capsule (TXL)	*Panax ginseng* C.A. Mey.; *Paeonia lactiflora* Pall.; Hirudo from *Hirudo nipponica* Whitman; *Buthus martensii* Karsch; *Scolopendra subspinipes mutilans* L. Koch; *Eupolyphaga sinensis* Walker; *Cryptotympana pustulata* Fabricius; *Ziziphus jujuba* Mill.; *Dalbergia odorifera* T.C. Chen; *Santalum album* L.; *Boswellia carterii* Birdw.; Borneolum Syntheticum	Ginsenoside Rb1, Rg1; paeoniflorin; hirudin-related peptides; saponins; volatile components	UPLC-Q-TOF-MS	CFDA 1996
Shexiang Tongxin Dropping Pill (STDP)	Synthetic Moschus; *Salvia miltiorrhiza* Bunge; Calculus Bovis Artifactus; Bufonis Venenum; Borneolum Syntheticum; total ginsenosides from *Panax ginseng* C.A. Mey. stems and leaves; Fel Ursi	Tanshinones; salvianolic acids; total ginsenosides; bufadienolides; bile acids; muscone (synthetic); 41 constituents identified	HPLC-Q-TOF-MS/MS	CFDA 2008
Tongmai Jiangtang Capsule (TJC)	*Pseudostellaria heterophylla* (Miq.) Pax; *Salvia miltiorrhiza* Bunge; *Coptis chinensis* Franch.; *Astragalus membranaceus* (Fisch.) Bunge; *Gynostemma pentaphyllum* (Thunb.) Makino; *Dioscorea opposita* Thunb.; *Atractylodes lancea* (Thunb.) DC.; *Scrophularia ningpoensis* Hemsl.; Hirudo; *Malva verticillata* L.; *Pueraria lobata* (Willd.) Ohwi	Berberine; astragaloside IV; puerarin; tanshinone IIA; salvianolic acid B	HPLC; quantitative quality control	NMPA approved
Compound Danshen Dropping Pill (CDDP)	*Salvia miltiorrhiza* Bunge; *Panax notoginseng* (Burkill) F.H. Chen; Borneolum Syntheticum	Tanshinol, salvianolic acids A/B/U/T/D; rosmarinic acid; protocatechualdehyde; notoginsenoside R1; ginsenosides Rb1/Rg1/Re; borneol	HPLC fingerprint	CFDA 1993; Chinese Pharmacopoeia

Botanical names in Table 4 are provided with author citations where available. Family names and medicinal parts should be interpreted according to pharmacopoeial source materials; for clarity, the principal plant-derived materials include Panax ginseng C.A. Mey. [Araliaceae; radix et rhizoma], Salvia miltiorrhiza Bunge [Lamiaceae; radix et rhizoma], Panax notoginseng (Burkill) F.H. Chen [Araliaceae; radix et rhizoma], Coptis chinensis Franch. [Ranunculaceae; rhizoma], Astragalus membranaceus (Fisch.) Bunge [Fabaceae; radix], Pueraria lobata (Willd.) Ohwi [Fabaceae; radix], Paeonia lactiflora Pall. [Paeoniaceae; radix], and Cinnamomum cassia Presl [Lauraceae; cortex]. Animal-derived, mineral-derived, resin, bile, venom, and synthetic components are retained at the pharmacopoeial material level.

**TABLE 5:** Compound-level mechanism mapping for representative TCM formulations.

Parent formula(s)	Source botanical/animal	Bioactive compound	Molecular target/pathway	Effect on microvascular biology	Tier	Module
SBP, TXL, STDP, CDDP	*Panax ginseng* C.A. Mey	Ginsenoside Rb1 (PubChem CID: 9898279)	PI3K/Akt → eNOS Ser1177; SIRT1/AMPK; CAT-1 (L-arginine)	Restores endothelial NO production; reverses H_2_O_2_-induced HUVEC senescence	4 + 5	Endo
SBP, TXL, STDP, CDDP	*Panax ginseng* C.A. Mey	Ginsenoside Rg1 (PubChem CID: 441923)	HIF-1α/VEGF; PI3K/Akt/mTOR; AMPK/PINK1	Promotes angiogenesis; preserves mitochondrial function under ischemia	4 + 5	Met, Rem
STDP, TJC, CDDP	*Salvia miltiorrhiza* Bunge	Tanshinone IIA (PubChem CID: 164676)	NOX4 inhibition; HSP90-eNOS coupling; ER/ERK1/2	Reverses high-glucose-induced eNOS uncoupling; restores endothelium-dependent vasodilation	4 + 5	Endo, IR
STDP, TJC, CDDP	*Salvia miltiorrhiza* Bunge	Salvianolic acid B (PubChem CID: 6451084)	PI3K/Akt-eNOS phosphorylation; AMPK Thr172; CAT-1/CAT-2B L-arginine uptake	Cardioprotective in MI model; vasodilation in eNOS−/− microvessels via compensatory pathways	4 + 5	Endo, IR
SBP	Moschus (*Moschus berezovskii* Flerov)	Muscone (PubChem CID: 7160826)	Connexin 43; vascular permeability; P-glycoprotein modulation	Modulates endothelial barrier function; “guide drug”	4 + 5	Endo, Rem
SBP, STDP	Bufonis Venenum from *Bufo gargarizans* Cantor	Bufalin (PubChem CID: 9547215); cinobufagin (PubChem CID: 11969542); resibufogenin (PubChem CID: 6917974)	Na^+^/K^+^-ATPase; Bcl-2/Bax; KATP channel	Positive inotropy; antiapoptotic in cardiomyocyte ischemia–reperfusion	5	Met
TXL	Hirudo (*Hirudo nipponica* Whitman)	Hirudin-related peptides (PubChem CID: Not applicable)	Direct thrombin (factor IIa) inhibition	Antithrombotic; improves microvascular rheology	4	Rem
TXL	*Paeonia lactiflora* Pall	Paeoniflorin (PubChem CID: 442534)	NF-κB p65; MAPK; TNF-α/IL-6 suppression	Attenuates endothelial inflammation and leukocyte adhesion	4 + 5	IR
TJC	*Astragalus membranaceus* (Fisch.) Bunge	Astragaloside IV (PubChem CID: 13943297)	AMPK; PI3K/Akt; eNOS phosphorylation	Improves endothelial function in db/db mouse aorta; insulin sensitization	4 + 5	Met, Endo
TJC	*Coptis chinensis* Franch	Berberine (PubChem CID: 2353)	AMPK; LDL receptor; gut microbiota	Glucose and lipid regulation; endothelial protection in hyperlipidemia	3 + 4	Met
TJC	*Pueraria lobata* (Willd.) Ohwi	Puerarin (PubChem CID: 5281807)	eNOS phosphorylation; NF-κB; Nrf2/HO-1	Antioxidant; vasodilation in ischemic heart models	3 + 4	Endo, IR
CDDP	*Panax notoginseng* (Burkill) F.H. Chen	Notoginsenoside R1 (PubChem CID: 441934)	PI3K/Akt; VEGF/HIF-1α; anti-platelet	Pro-angiogenic in MI model; improves microcirculation	4	Rem
All formulas	Borneolum Syntheticum	Borneol (PubChem CID: 64685)	Tight-junction modulation; P-glycoprotein modulation	“Envoy drug” — facilitates penetration of co-administered compounds	4	Endo

PubChem compound identifiers (CID) are provided for principal small-molecule constituents when available. Tier refers to the evidence hierarchy in Table 2. Microvascular module: Endo = endothelial-vasomotor; IR, inflammatory-redox; Met = metabolic-mitochondrial; Rem = structural remodeling; HUVEC, human umbilical vein endothelial cell; MI, myocardial infarction; TCM, traditional Chinese medicine.

### 5.1 Shexiang Baoxin Pill (SBP)

Shexiang Baoxin Pill (SBP) is a Chinese patent medicine listed in the Chinese Pharmacopoeia (2015 edition). Its composition includes Moschus, Panax ginseng C.A. Mey., Calculus Bovis Artifactus, Cinnamomum cassia Presl, Styrax from Liquidambar orientalis Mill., Bufonis Venenum from *Bufo gargarizans* Cantor, and Borneolum Syntheticum. More than seventy non-volatile and forty volatile constituents have been identified by HPLC and GC-MS (Lu et al., 2018; Wang M. et al., 2021).

Mechanistically, characterized SBP constituents engage multiple modules of microvascular biology described in Section 3. Ginsenoside Rb1 enhances eNOS Ser1177 phosphorylation through PI3K/Akt and engages SIRT1/AMPK signaling; ginsenoside Rg1 promotes HIF-1α–VEGF–driven angiogenesis and supports mitochondrial quality control through AMPK/PINK1; muscone modulates connexin 43 and microvascular permeability and acts as a “guide” to facilitate penetration of co-administered constituents; bufadienolides influence Na^+^/K^+^-ATPase and apoptosis regulators; borneol modulates tight-junction permeability. These mechanisms have been reported in cell culture, isolated microvessels, and animal models of myocardial ischemia–reperfusion or hyperglycemic vascular injury (Tier 4–5 evidence) (Lu et al., 2018; Wang M. et al., 2021).

Critically, SBP is the only formulation reviewed here for which Tier 1 ANOCA evidence is currently available. This phase IV ANOCA trial—a multicenter, randomized, double-blind, placebo-controlled study—enrolled 239 patients with ANOCA at 11 centers in China between 2021 and 2023, with treatment for 12 weeks (He et al., 2025). The primary outcome was change in the Seattle Angina Questionnaire score, and the trial reported significant improvement in angina-related quality of life with SBP versus placebo, with a safety profile comparable to placebo. This is the first phase IV randomized evidence in a phenotype-defined ANOCA population for any TCM formulation. Earlier evidence in stable coronary artery disease (the larger MUSKARDIA (MUST) trial in 2,674 patients) showed no significant reduction in major adverse cardiovascular events at 24 months, although subgroup signals favored women and patients with BMI < 24 kg/m^2^ (Ge et al., 2021). A separate meta-analysis of SBP for coronary microvascular function (Wang M. et al., 2021) was classified as Tier 3, as it pooled data from stable CAD and microvascular angina cohorts without requiring invasive phenotypic confirmation for inclusion.

Calibrated to the available evidence, SBP has demonstrated benefit on patient-reported angina outcomes in ANOCA in a single phase IV trial; the effect is supported by mechanistic plausibility across all four microvascular biology modules; and confirmation in an independent ANOCA cohort with invasive microvascular phenotyping (CFR, IMR) remains a priority for the field.

Target–clinical linkage for SBP. Among the reported mechanisms, the eNOS/NO–CFR connection is best supported (validated), as the phase IV trial (He et al., 2025) assessed angina symptoms alongside functional microvascular indices. The anti-inflammatory effects (NF-κB/hs-CRP) and metabolic modulation (AMPK/glucose-lipid profile) are supported by biomarker data from secondary analyses (partially validated). By contrast, the HIF-1α/VEGF–angiogenesis pathway and mitochondrial effects rest exclusively on preclinical data and are therefore considered speculative with respect to clinical translation in ANOCA.

### 5.2 Tongxinluo Capsule (TXL)

Tongxinluo Capsule (TXL) was developed under the modern collateral-disease framework and approved by the China Food and Drug Administration in 1996. Its composition includes Panax ginseng C.A. Mey., Paeonia lactiflora Pall., Hirudo from Hirudo nipponica Whitman, Buthus martensii Karsch, Scolopendra subspinipes mutilans L. Koch, Eupolyphaga sinensis Walker, Cryptotympana pustulata Fabricius, Ziziphus jujuba Mill., Dalbergia odorifera T.C. Chen, Santalum album L., Boswellia carterii Birdw., and Borneolum Syntheticum. UPLC-Q-TOF-MS profiling has identified saponins, flavonoids, peptides, and volatile constituents (Xin et al., 2024).

Mechanistic studies point to several relevant activities: hirudin-related thrombin inhibition and improved microvascular rheology; paeoniflorin-mediated suppression of NF-κB activation and reduction of endothelial leukocyte adhesion; ginsenoside-mediated effects on the eNOS/NO axis and PI3K/Akt; and induction of autophagy through MEK/ERK in cardiac microvascular endothelial cells exposed to hypoxia/reoxygenation. Animal models of myocardial ischemia and microvascular injury support these mechanisms (Tier 4) (Xin et al., 2024).

Clinical evidence in ANOCA-defined populations is limited; most TXL clinical trials have been conducted in stable coronary artery disease, microvascular angina, or post-percutaneous coronary intervention “no-reflow” — populations that overlap with but are not identical to ANOCA. These constitute Tier 2 to Tier 3 evidence. Several meta-analyses (Tier 3) have reported improvements in angina frequency, exercise capacity, and ECG indices with TXL added to standard therapy. However, the included trials are predominantly Chinese-language single-center studies in stable CAD or post-PCI populations, and none of the source cohorts required invasive microvascular phenotyping (CFR, IMR, or acetylcholine provocation) for study entry. Methodological heterogeneity across these studies limits the interpretability of pooled estimates.

Taken together, the available data indicate that TXL has biologically plausible preclinical activity across endothelial, inflammatory, and rheological modules; clinical evidence in formally defined ANOCA cohorts is currently absent. A randomized trial in ANOCA with invasive microvascular endpoints would clarify the formulation’s clinical position.

Target–clinical linkage for TXL. The anti-inflammatory (NF-κB/hs-CRP) and antithrombotic (hirudin) mechanisms have been validated in stable CAD cohorts at Tier 3 (partially validated). The eNOS/NO–CFR link is inferred from ginsenoside studies but has not been confirmed in TXL-treated ANOCA patients (speculative). Preclinical findings on autophagy and angiogenesis remain without clinical correlate (speculative).

### 5.3 Shexiang Tongxin Dropping Pill (STDP)

Shexiang Tongxin Dropping Pill (STDP) is a Chinese patent medicine approved by the China Food and Drug Administration in 2008. Its composition includes synthetic Moschus, Salvia miltiorrhiza Bunge, Calculus Bovis Artifactus, Bufonis Venenum, Borneolum Syntheticum, total ginsenosides from Panax ginseng C.A. Mey. stems and leaves, and Fel Ursi. Forty-one constituents—including bile acids, salvianolic acids, triterpene saponins, bufadienolides, and tanshinones—have been identified by HPLC-Q-TOF-MS/MS (Chen et al., 2015; Cui et al., 2024).

Of direct relevance to coronary microvascular dysfunction, Cui and colleagues reported that STDP promotes angiogenesis through the VEGF/eNOS pathway in a model of diabetic coronary microcirculation dysfunction, with histologically demonstrated improvements in capillary density and functional improvement in coronary flow indices (Tier 4 evidence) (Cui et al., 2024). Salvianolic acids and tanshinone IIA—both characterized constituents—engage the eNOS axis and Nrf2/HO-1 antioxidant signaling.

Clinical evidence is at Tier 3: STDP is registered for use in chronic coronary disease and angina with blood-stasis features, with reported improvements in angina frequency and ECG indices. ANOCA-specific randomized evidence is not currently available.

Considered against the evidence hierarchy, STDP shows mechanistically plausible preclinical activity in microvascular angiogenesis through a pathway central to ANOCA biology, but has not yet been tested in phenotype-defined ANOCA cohorts.

Target–clinical linkage for STDP. The VEGF/eNOS–angiogenesis mechanism, demonstrated in diabetic microvascular models (Cui et al., 2024), corresponds clinically to capillary density and coronary flow reserve, but these indices have not been measured in STDP-treated patients. The Nrf2/HO-1 antioxidant pathway is supported by preclinical data but lacks clinical biomarker correlation. Thus, all mechanism–endpoint connections for STDP remain speculative (Tier 4–5), pending ANOCA-specific clinical trials with invasive or imaging-based microvascular endpoints.

### 5.4 Tongmai Jiangtang Capsule (TJC)

Tongmai Jiangtang Capsule (TJC) is a Chinese patent medicine registered with the National Medical Products Administration for diabetic vascular complications. Its composition includes Pseudostellaria heterophylla (Miq.) Pax, Salvia miltiorrhiza Bunge, Coptis chinensis Franch., Astragalus membranaceus (Fisch.) Bunge, Gynostemma pentaphyllum (Thunb.) Makino, Dioscorea opposita Thunb., Atractylodes lancea (Thunb.) DC., Scrophularia ningpoensis Hemsl., Hirudo, Malva verticillata L., and Pueraria lobata (Willd.) Ohwi. Quality-controlled active constituents include berberine, astragaloside IV, puerarin, tanshinone IIA, and salvianolic acid B (Wan et al., 2025; Wang Y. et al., 2021).

The formulation is of particular interest for the cardiometabolic-inflammatory phenotype of ANOCA seen in patients with type 2 diabetes. Berberine engages AMPK and improves endothelial function in hyperlipidemic and hyperglycemic settings (Su et al., 2026); astragaloside IV activates AMPK and improves endothelial function in db/db mouse aorta; puerarin modulates eNOS phosphorylation and Nrf2/HO-1; salvianolic acid B and tanshinone IIA engage the eNOS axis. The convergence of these constituents on metabolic, redox, and endothelial-vasomotor modules is mechanistically rational for the cardiometabolic ANOCA subtype.

Clinical evidence in ANOCA *per se* is absent; TJC has been studied in type 2 diabetes mellitus complicated by coronary heart disease, with reported improvements in angina symptoms, glycemic indices, and inflammatory markers (Wan et al., 2025; Wang Y. et al., 2021). This is Tier 3 evidence in an overlapping but distinct population.

On the basis of available evidence, TJC has a mechanistically plausible constituent profile for diabetic coronary microvascular dysfunction; whether the pattern of benefit extends to non-diabetic ANOCA, and whether it includes invasive microvascular endpoints, has not yet been tested.

Target–clinical linkage for TJC. The AMPK–metabolic (glucose/lipid) connection is validated in diabetic cohorts (Wan et al., 2025; Tier 3). The eNOS/NO and Nrf2/HO-1 pathways are supported by biomarker changes (e.g., NO, hs-CRP) in the same studies (partially validated). The anti-inflammatory effects on NF-κB/cytokines remain at the preclinical level and have not been separately confirmed in clinical specimens (speculative).

### 5.5 Compound Danshen Dropping Pill (CDDP)

Compound Danshen Dropping Pill (CDDP) is a Chinese patent medicine approved by the China Food and Drug Administration in 1993 and listed in the Chinese Pharmacopoeia. It contains Salvia miltiorrhiza Bunge, Panax notoginseng (Burkill) F.H. Chen, and Borneolum Syntheticum. Pharmacopoeia-defined active fractions include phenolic acids (tanshinol, salvianolic acids A, B, U, T, D, rosmarinic acid, protocatechualdehyde) and saponins (notoginsenoside R1, ginsenosides Rb1, Rg1, Re).

Mechanistic activity is concentrated on the endothelial-vasomotor and inflammatory-redox modules. Tanshinone IIA reverses high-glucose-induced eNOS uncoupling; salvianolic acid B promotes eNOS phosphorylation through PI3K/Akt and supports L-arginine substrate availability; notoginsenoside R1 promotes angiogenesis through HIF-1α/VEGF and reduces platelet activation. Animal models of myocardial ischemia–reperfusion and microvascular injury have repeatedly shown these effects (Tier 4) (Horton and Barrett, 2021).

Clinical evidence is largely at Tier 3. One meta-analysis of blood-activating TCM formulas (Zhong et al., 2020) reported reductions in angina frequency and improvements in microcirculatory biomarkers (NO, ET-1, hs-CRP). Most included trials enrolled patients with chronic stable angina or clinically defined microvascular angina without invasive confirmation of coronary microvascular dysfunction (CFR, IMR, or acetylcholine provocation); thus the pooled evidence was classified as Tier 3. The few constituent studies that did use invasive CFR measurement were considered Tier 2 on an individual basis, but they are too few and too heterogeneous to support a formulation-specific conclusion.

Overall, CDDP has a chemically simpler profile than the other formulations reviewed and a correspondingly more focused mechanistic rationale centered on the eNOS-NO axis and adaptive angiogenesis; ANOCA-specific clinical confirmation remains the principal evidence gap.

Target–clinical linkage for CDDP. The eNOS/NO–CFR connection is supported by biomarker data (NO, ET-1) from microvascular angina trials (Zhong et al., 2020) (partially validated, Tier 3), though CFR was not directly measured. The HIF-1α/VEGF–angiogenesis pathway rests exclusively on animal models and is therefore speculative (Tier 4). ANOCA-specific clinical confirmation with invasive microvascular endpoints remains the principal evidence gap.

## 6 Critical limitations and translational challenges

The evidence base reviewed above has several limitations that should constrain interpretation.

### 6.1 Phenotype heterogeneity

ANOCA is a heterogeneous syndrome that includes microvascular spasm, endothelium-dependent and endothelium-independent CFR abnormalities, and combinations thereof, alongside the coronary artery spasm phenotype that overlaps clinically. Most published TCM trials have not stratified by microvascular phenotype, and only a minority have used invasive testing (CFR, IMR, acetylcholine provocation) as an inclusion or outcome criterion. Without phenotype stratification, between-trial comparison is constrained.

### 6.2 Chemical heterogeneity

Multi-component formulations vary in constituent profile across manufacturing batches, particularly when biological materials (e.g., Bufonis Venenum, Hirudo) are involved. Trials and mechanistic studies that do not report chemical fingerprinting limit reproducibility. Pharmacopoeial standardization addresses this in principle, but compliance varies.

### 6.3 Trial methodology

A substantial proportion of the supportive clinical literature consists of single-center Chinese-language trials with small samples, short follow-up, surrogate endpoints, and inconsistent blinding. Meta-analyses can mask the resulting heterogeneity. The phase IV ANOCA trial of Shexiang Baoxin Pill is a notable methodological exception (He et al., 2025), but it remains the only ANOCA-specific phase IV trial in this drug class.

### 6.4 Mechanism–endpoint distance

Many mechanistic studies report effects on intermediate biomarkers (NO, ET-1, hs-CRP, ICAM-1) rather than on phenotype-defined microvascular function. Whether biomarker change translates to functional or symptomatic improvement is not always clear and requires direct testing.

### 6.5 Evidence-tier confusion

As noted throughout this review, the TCM literature has historically conflated evidence tiers. Statements in the present review are calibrated to the relevant tier, but the literature outside this review is not, and clinicians evaluating TCM evidence should attend to whether a given claim is supported by Tier 1 or Tier 5 evidence.

### 6.6 Regulatory and integration considerations

The pharmacological co-administration of TCM with standard antianginal therapy (calcium channel blockers, beta-blockers, statins, antiplatelet agents) is the rule rather than the exception in clinical practice in China. Drug–drug interactions, particularly involving CYP-mediated metabolism and P-glycoprotein, are not always systematically reported in TCM trials and represent a gap in the safety evidence base.

## 7 Future directions

Several research priorities emerge from this synthesis.

### 7.1 Phenotype-defined ANOCA cohorts

Future TCM trials should adopt the invasive microvascular phenotyping framework advanced by the COVADIS criteria—coronary flow reserve, index of microcirculatory resistance, acetylcholine provocation—as inclusion criteria, outcome measures, or both. This is the methodological standard that the broader ANOCA field has now adopted, and it is the standard that will determine whether TCM findings can be considered for integration into international cardiology practice.

### 7.2 Compound-level dose–response

Where possible, mechanistic claims about formulations should be supported by dose–response data on individual characterized constituents, with confirmation of the mechanism in cell models, animal models, and (for the most advanced formulations) in human pharmacokinetic-pharmacodynamic studies.

### 7.3 Standardized chemical fingerprinting

Every clinical trial of a multi-component TCM formulation in cardiovascular disease should report HPLC or LC-MS fingerprint data, with quantification of the principal characterized bioactive constituents in the trial product, so that the result is interpretable and reproducible.

### 7.4 Hypothesis-driven syndrome–phenotype trials

The relationship between TCM syndrome patterns and microvascular phenotypes is currently a heuristic. Prospective trials in which patients are stratified both by TCM syndrome and by microvascular phenotype, and in which the formulation is selected accordingly, would test whether syndrome differentiation has clinical utility beyond a tradition-based stratification tool.

### 7.5 Drug–drug interaction profiling

Systematic profiling of CYP and P-glycoprotein interactions for the principal bioactive constituents of each formulation, together with formal interaction studies in patients on standard antianginal therapy, would close a current gap in the safety evidence base.

### 7.6 Open data and reporting

Adoption of the relevant CONSORT extensions for herbal interventions (CONSORT-Chinese Herbal Formulas) and full reporting of chemical, pharmacological, and clinical methodology would substantially raise the quality of new TCM evidence in this field.

## 8 Conclusion

Coronary microvascular dysfunction underlying ANOCA is a multi-mechanism disorder for which single-target therapy has been structurally insufficient. The pharmacology of representative TCM formulations is best considered multi-target by chemical structure and, in modern systems-pharmacology terms, appears to distribute activity across the endothelial-vasomotor, inflammatory-redox, metabolic-mitochondrial, and structural-remodeling modules that define the disease. At the level of bioactive constituents, the activity is increasingly traceable to specific compounds and specific molecular targets, although the clinical significance of many of these targets remains to be validated in ANOCA-specific cohorts.

The clinical position of these formulations in ANOCA, however, is uneven. The phase IV ANOCA trial of Shexiang Baoxin Pill is the only randomized clinical evidence currently available in a phenotype-defined ANOCA population. The remaining formulations rest on a base of mechanistic plausibility and adjacent clinical evidence in stable coronary disease, microvascular angina, or diabetic coronary complications, and lack adequately powered ANOCA-specific randomized trials.

This review therefore provides an evidence-calibrated framework: an explicit hierarchy that distinguishes Tier 1 evidence from Tier 5; a constituent-level rather than formulation-level mechanistic accounting; and a clear separation between hypothesis-generating elements (such as syndrome–phenotype mapping) and evidence-supported elements (such as the microvascular benefit demonstrated in the phase IV ANOCA trial). The path from this synthesis to clinical translation is straightforward in outline—phenotype-defined cohorts, standardized formulations, prespecified microvascular endpoints—but it is not yet the standard in the field. Whether TCM enters the evidence-based therapeutic vocabulary for ANOCA will depend on whether the field meets that standard.

## Glossary

AMPK: AMP-activated protein kinase
ANOCA: angina with non-obstructive coronary arteries
CAD: coronary artery disease
CDDP: Compound Danshen Dropping Pill
CFR: coronary flow reserve
CMD: coronary microvascular dysfunction
eNOS: endothelial nitric oxide synthase
ET-1: endothelin-1
GC-MS: gas chromatography-mass spectrometry
HIF-1α: hypoxia-inducible factor 1-alpha
HO-1: heme oxygenase-1
HPLC: high-performance liquid chromatography
HUVEC: human umbilical vein endothelial cell
ICAM-1: intercellular adhesion molecule-1
IMR: index of microcirculatory resistance
INOCA: ischemia with non-obstructive coronary arteries
LC-MS: liquid chromatography-mass spectrometry
MI: myocardial infarction
NADPH: nicotinamide adenine dinucleotide phosphate
NF-κB: nuclear factor kappa-B
NOX: NADPH oxidase
Nrf2: nuclear factor erythroid 2-related factor 2
PI3K: phosphoinositide 3-kinase
PINK1: PTEN-induced putative kinase 1
ROS: reactive oxygen species
SAQ: Seattle Angina Questionnaire
SBP: Shexiang Baoxin Pill
SIRT1: sirtuin 1
SOD: superoxide dismutase
STDP: Shexiang Tongxin Dropping Pill
T2DM: type 2 diabetes mellitus
TCM: traditional Chinese medicine
TGF-β: transforming growth factor beta
TJC: Tongmai Jiangtang Capsule
TNF-α: tumor necrosis factor alpha
TXL: Tongxinluo Capsule
UPLC-Q-TOF-MS: ultra-performance liquid chromatography-quadrupole time-of-flight mass spectrometry
VCAM-1: vascular cell adhesion molecule-1
VEGF: vascular endothelial growth factor.
